# Minimally invasive or sternotomy approach in mitral valve surgery: a propensity-matched comparison

**DOI:** 10.1186/s13019-021-01578-9

**Published:** 2021-08-10

**Authors:** Marek Pojar, Mikita Karalko, Martin Dergel, Jan Vojacek

**Affiliations:** Department of Cardiac Surgery, Faculty of Medicine in Hradec Kralove and University Hospital Hradec Kralove, Charles University, Sokolska 581, 500 05 Hradec Kralove, Czech Republic

**Keywords:** Minimally invasive, Minithoracotomy, Mitral valve, Endoscopic surgery

## Abstract

**Objectives:**

Conventional mitral valve surgery through median sternotomy improves long-term survival with acceptable morbidity and mortality. However, less-invasive approaches to mitral valve surgery are now increasingly employed. Whether minimally invasive mitral valve surgery is superior to conventional surgery is uncertain.

**Methods:**

A retrospective analysis of patients who underwent mitral valve surgery via minithoracotomy or median sternotomy between 2012 and 2018. A propensity score-matched analysis was generated to eliminate differences in relevant preoperative risk factors between the two groups.

**Results:**

Data from 525 patients were evaluated, 189 underwent minithoracotomy and 336 underwent median sternotomy. The 30 day mortality was similar between the minithoracotomy and conventional surgery groups (1 and 3%, respectively; *p* = 0.25). No differences were seen in the incidence of stroke (*p* = 1.00), surgical site infections (*p* = 0.09), or myocardial infarction (*p* = 0.23), or in total hospital cost (*p* = 0.48). However, the minimally invasive approach was associated with fewer patients receiving transfusions (59% versus 76% in the conventional group; *p* = 0.001) or requiring reoperation for bleeding (3% versus 9%, respectively; *p* = 0.03). There were no significant differences in 5 year survival between the minithoracotomy and conventional surgery groups (93% versus 86%, respectively; *p* = 0.21) and freedom from mitral valve reoperation (95% versus 94%, respectively; *p* = 0.79).

**Conclusions:**

In patients undergoing mitral valve surgery, a minimally invasive approach is feasible, safe, and reproducible with excellent short-term outcomes; mid-term outcomes and efficacy were also seen to be comparable to conventional sternotomy.

## Introduction

Minimally invasive cardiac surgery has become increasingly popular, and several techniques for minimally invasive mitral valve surgery (MIMVS) have been developed in recent decades, and have gained increasing acceptance. Right minithoracotomy, with the use of video assistance or complete video control during surgery is now the most accepted and widespread approach.

Clinical studies have shown excellent results for MIMVS compared with conventional sternotomy, in terms of a reduction in morbidity, surgical trauma, pain, and shorter hospital stay, as well as enabling faster recovery, an earlier return to full activities, superior preservation of lung function, and improved cosmetic results. However, MIMVS is associated with longer surgery and cardiopulmonary bypass times [1, 2]. Propensity score analysis is often used in retrospective studies to control for treatment allocation bias, which is inherent in retrospective studies. In the present study, a propensity-matched comparison was carried out to compare short- and mid-term outcomes in patients who underwent MIMVS with those who underwent conventional full sternotomy. This study also evaluated the healthcare costs associated with a minimally invasive approach relative to a traditional surgery.

## Materials and methods

Data from patients who underwent isolated mitral valve surgery with or without tricuspid valve repair and cryoablation between January 2012 and December 2018 were retrospectively evaluated. Patients undergoing concomitant aortic valve surgery, coronary artery bypass grafting, or reoperation were excluded, as were those with endocarditis or patients undergoing emergency procedures. All operations were performed by senior surgeons experienced in mitral valve and minimally invasive surgery. Patients were grouped according to the type of surgery, i.e., minimally invasive minithoracotomy (MINI group) or conventional full sternotomy (STERNOTOMY group). Contra-indications for a minimally invasive approach were as follows: dilated ascending aorta (> 40 mm), aortic regurgitation > grade 1, severe peripheral vascular disease, ascending aorta calcifications, right lung operation, and an expectation of right pleural cavity adhesions.

Data were retrieved from the prospective National Cardiac Surgery registry and from the patients’ medical records. Complete operative and postoperative costs associated with mitral valve surgery were individually gathered from institutional billing records. Only direct hospital costs were used in this analysis. Postoperative outcomes and major complications were also analyzed.

All patients underwent preoperative transesophageal echocardiography as well as transthoracic echocardiography at discharge. The severity of mitral valve regurgitation was graded according to the recommendations of the European Society of Cardiology and the European Association for Cardio-Thoracic Surgery [3]. Echocardiographic follow-up was conducted in all survivors who had received a postoperative echocardiogram > 3 months after surgery. Follow-up data on survival and reoperation were collected from the hospital or from the health insurance database, and supplementary information was supplied from referring cardiologists and family physicians.

The study design complied with the Declaration of Helsinki, and the Ethical committee of University Hospital Hradec Kralove approved the study (reference number: 201902 S19P). The requirement for individual patient consent was waived due to the retrospective nature of the study. Signed informed consent for the surgical procedure was obtained from each patient.

### Surgical technique

Conventional general anesthesia was used in all patients regardless of surgical approach. In the sternotomy group, median sternotomy and pericardial opening were performed in a standard manner. Cardiopulmonary bypass was established with double venous and aortic cannulation, and cold blood intermittent cardioplegia delivered in an antegrade fashion. Routine mitral valve repair and replacement techniques were used in both surgical approaches. Patients undergoing a minimally invasive approach were intubated with a single lumen endotracheal tube. After general anesthesia was initiated, the superior vena cava was cannulated percutaneously (Fem-FlexII, Edwards Lifesciences Inc., Irvine, CA, USA) via the right jugular vein to obtain adequate venous return. The standard surgical approach for MIMVS has been reported elsewhere [2]. Briefly, MIMVS, by way of right minithoracotomy, was performed through a 5–7 cm skin incision at the 4th intercostal space. After skin incision, a soft tissue retractor (ValveGate Soft Tissue Retractor; Geister, Tuttlingen, Germany) was inserted, and the intercostal space was gently spread. Additional small (5–10 mm) incisions were used for video assistance, the left atrial retractor, and the transthoracic aortic clamp. The right femoral vessels were exposed through a transverse incision, and a venous cannula (QuickDraw; Edwards Lifesciences Inc., Irvine, CA, USA) was inserted through the femoral vein into the right atrium. Correct positioning was achieved with the Seldinger technique under transoesophageal echocardiographic guidance. The femoral artery was cannulated using an arterial cannula (Fem-FlexII, Edwards Lifesciences Inc.). After vacuum-assisted cardiopulmonary bypass (− 40 to − 60 mmHg) was established, patients were cooled to 34 °C, and the ascending aorta was clamped with a Chitwood clamp. An antegrade cold crystalloid cardioplegia (Custodiol-CE; Dr. Franz Köhler Chemie, Bensheim, Germany) was delivered directly into the ascending aorta by a needle vent catheter. The surgical field was constantly flushed with CO_2_ through the camera port. The mitral valve was approached with a traditional left paraseptal atriotomy and exposed using a specially designed atrial retractor. A video camera was placed through a 5 mm port in the 4th intercostal space. The procedure was performed under direct vision with video assistance.

Decisions regarding intraoperative transfusion, anesthetic technique, and the timing of extubation were made at the discretion of the anesthesiologist. Intraoperative and postoperative transfusion, extubation, and intensive care unit management were not derived from protocols.

### Statistical analysis

Continuous variables are expressed as mean ± standard deviation (median, interquartile range), and categorical data are presented as frequencies and percentages. Differences between groups were assessed using Fisher’s exact test for categorical variables and the Mann–Whitney *U*-test or Kolmogorov–Smirnov test for non-normally distributed continuous variables. The differences between the MINI and STERNOTOMY groups limited direct comparison of the patients. To compensate for these differences, propensity score-matched groups were generated based on variables including demographics, comorbidities, and intraoperative data. Covariates included in calculation of the propensity score included age at surgery, acute operation, EuroScore II, creatinine concentration, NYHA class, mitral valve stenosis and regurgitation, mitral valve regurgitation grade, tricuspid valve operation, and comorbidities such as diabetes mellitus, peripheral arterial disease and rheumatic disease. The patients were matched using a maximum 1:2 greedy nearest-neighbor algorithm with a maximum caliper width of 0.05. Confounders included in the propensity score model were compared between treatment groups before and after matching. The analysis was performed on an intention-to-treat basis, meaning patients assigned to undergo a minimally invasive approach who were converted to sternotomy were included in the minimally invasive group. Statistical analysis was performed with NCSS 11 Statistical Software 2016 (NCSS, LLC, Kaysville, UT, USA). Survival and freedom from reoperation were estimated with the standard nonparametric Kaplan–Meier method, and compared using a log-rank test. A *p*-value ≤0.05 was considered statistically significant.

## Results

### Preoperative variables

A total of 525 patients underwent mitral valve surgery with or without tricuspid valve repair and cryoablation during the study period; 189 (36%) were performed by a minimally invasive minithoracotomy and 336 (64%) by conventional full median sternotomy. The characteristics of the overall study population are summarized in Table 1. Patients in the MINI group were more likely to be younger (64.1 ± 9.1 versus 65.6 ± 10.4 in the STERNOTOMY group; *p* = 0.02), less symptomatic in terms of NYHA class III + IV (33% versus 54%, respectively; *p* <  0.001), and have fewer comorbidities, reflected by a lower EuroScore II value (2.4 ± 2.2 versus 4.1 ± 5.2, respectively; *p* <  0.001). Fewer patients in the MINI group underwent acute operation (0% versus 5% in the STERNOTOMY group; *p* <  0.001), and fewer underwent surgery because of mitral valve stenosis (1% versus 8%, respectively; *p* <  0.001).

**Table 1:** Characteristics of the overall population and propensity matched patients

	Overall			Matched Patients		
	MINI	STERNOTOMY	*p*-value	MINI	STERNOTOMY	*p*-value
Variable	*n* = 189	*n* = 336		*n* = 158	*n* = 225	
Age (years)	64.1 ± 9.1	65.6 ± 10.4	**0.02**	64.8 ± 8.9	65.4 ± 10.9	0.16
Gender (male)	85 (45%)	145 (43%)	0.71	64 (41%)	95 (42%)	0.75
BMI (kg/m^2^)	27.9 ± 4.4	29.0 ± 6.1	0.11	28.0 ± 4.5	29.3 ± 6.2	0.07
Previous MI	12 (6%)	35 (10%)	0.15	8 (5%)	20 (9%)	0.17
Diabetes mellitus	23 (12%)	76 (23%)	**0.004**	22 (14%)	39 (17%)	0.40
Arterial hypertension	136 (72%)	239 (71%)	0.23	119 (75%)	161 (72%)	0.35
COPD	23 (12%)	48 (14%)	0.60	20 (13%)	25 (11%)	0.75
Hyperlipidaemia	95 (50%)	179 (53%)	0.53	83 (53%)	117 (52%)	1.00
Atrial fibrillation	77 (41%)	148 (44%)	0.52	71 (45%)	99 (44%)	0.92
Peripheral arterial disease	1 (1%)	12 (4%)	**0.04**	1 (1%)	1 (0%)	1.00
Cerebrovascular disease	16 (8%)	24 (7%)	0.61	16 (10%)	15 (7%)	0.26
Smoking	49 (26%)	101 (30%)	0.37	39 (25%)	54 (24%)	0.90
LVEF (%)	58.2 ± 11.3	57.3 ± 12.0	0.10	58.9 ± 10.4	58.1 ± 11.0	0.15
Pulmonary hypertension	21 (11%)	57 (17%)	0.08	21 (13%)	31 (14%)	1.00
EuroScore II (%)	2.4 ± 2.2	4.1 ± 5.2	**< 0.001**	2.6 ± 2.3	2.9 ± 2.2	0.06
Creatinine concentration (μmol/l)	83.0 ± 18.6	99.4 ± 59.9	**< 0.001**	83.7 ± 19.2	87.5 ± 21.2	0.10
NYHA class						
I + II	126 (67%)	144 (43%)	**< 0.001**	95 (60%)	122 (54%)	0.30
III + IV	63 (33%)	180 (54%)	**< 0.001**	63 (40%)	103 (46%)	0.30
Mitral valve stenosis	2 (1%)	27 (8%)	**< 0.001**	2 (1%)	6 (3%)	0.48
Mitral valve regurgitation	187 (99%)	309 (92%)	**< 0.001**	156 (99%)	219 (97%)	0.48
Mitral valve regurgitation grade						
1 + 2	1 (1%)	15 (4%)	**0.01**	1 (1%)	5 (2%)	0.41
3 + 4	188 (99%)	315 (94%)	**< 0.001**	157 (99%)	217 (96%)	0.09
Mitral valve pathology						
Degenerative	166 (88%)	281 (84%)	0.20	140 (89%)	203 (90%)	0.62
Rheumatic	1 (1%)	14 (4%)	**0.01**	1 (1%)	3 (1%)	0.65
Ischemic	11 (6%)	23 (7%)	0.72	7 (4%)	11 (5%)	1.00
Secondary	11 (6%)	13 (4%)	0.38	10 (6%)	8 (4%)	0.23
Acute operation	0 (0%)	16 (5%)	**< 0.001**	0 (0%)	0 (0%)	1.00

*BMI* body mass index; *COPD* chronic obstructive pulmonary disease; *LVEF* left ventricle ejection fraction; Pulmonary hypertension –systolic pulmonary artery pressure ≥ 55 mmHg

Using a matching technique, 158 patients in the MINI group and 225 patients in the STERNOTOMY group were included in the additional analysis. Matched groups were similar with regard to all preoperative comorbidity, mitral valve pathology, and demographic categories (Table 1).

### Operative and postoperative results

The surgical data and postoperative results are described in Table 2. As expected, the duration of surgery (232.0 ± 44.5 versus 226.4 ± 66.0; *p* = 0.01), cardiopulmonary bypass (145.6 ± 31.9 versus 110.7 ± 35.9; *p* <  0.001), and cross-clamping (97.7 ± 25.4 versus 84.8 ± 28.0; *p* <  0.001) was significantly longer in the MINI group than the STERNOTOMY group. However, this did not lead to differences in the short-term outcomes, as 30 day mortality was 1% versus 3% (*p* = 0.25) for minimally invasive and sternotomy patients, respectively.

**Table 2:** Intra- and post-operative results of the overall population and propensity matched patients

	Overall			Matched Patients		
	MINI	STERNOTOMY	*p*-value	MINI	STERNOTOMY	*p*-value
Variable	*n* = 189	*n* = 336		*n* = 158	*n* = 225	
CPB time (minutes)	141.2 ± 32.6	111.8 ± 33.8	**< 0.001**	145.6 ± 31.9	110.7 ± 35.9	**< 0.001**
Cross-clamp time (minutes)	94.4 ± 25.4	85.0 ± 26.1	**< 0.001**	97.7 ± 25.4	84.8 ± 28.0	**< 0.001**
Operative time (minutes)	227.6 ± 44.0	227.8 ± 62.3	0.39	232.0 ± 44.5	226.4 ± 66.0	**0.01**
Concomitant procedure						
Tricuspid annuloplasty	73 (39%)	186 (55%)	**< 0.001**	73 (46%)	116 (52%)	0.35
Cryoablation	83 (44%)	157 (47%)	0.58	74 (47%)	105 (47%)	1.00
MI	6 (3%)	6 (2%)	0.35	6 (4%)	4 (2%)	0.23
Renal replacement therapy	4 (2%)	21 (6%)	**0.03**	4 (3%)	6 (3%)	1.00
Cerebrovascular stroke and TIA	6 (3%)	12 (4%)	1.00	5 (3%)	6 (3%)	0.77
Stroke	4 (2%)	10 (3%)	0.59	3 (2%)	4 (2%)	1.00
Delirium	22 (12%)	30 (9%)	0.36	19 (12%)	17 (8%)	0.16
Pneumonia	10 (5%)	33 (10%)	0.10	9 (6%)	19 (8%)	0.43
Re-exploration for bleeding	8 (4%)	35 (10%)	**0.01**	5 (3%)	20 (9%)	**0.03**
Blood loss / 24 h (ml)	668.8 ± 519.7	872.9 ± 865.6	**< 0.001**	661.7 ± 466.8	832.9 ± 852.0	**0.02**
Transfusion	106 (56%)	259 (77%)	**< 0.001**	94 (59%)	170 (76%)	**0.001**
Number of Transfusions	2.5 ± 4.3	5.3 ± 7.1	**< 0.001**	2.7 ± 4.2	4.6 ± 6.3	**< 0.001**
Atrial fibrillation	74 (39%)	142 (42%)	0.52	63 (40%)	89 (40%)	1.00
Wound infection	1 (1%)	8 (2%)	0.17	1 (1%)	8 (4%)	0.09
Low output syndrome	9 (5%)	40 (12%)	**0.007**	8 (5%)	21 (9%)	0.17
Time to extubation (hour)	12.5 ± 13.3	29.6 ± 88.9	**0.001**	13.0 ± 14.2	20.9 ± 63.2	0.26
Prolonged ventilation	11 (6%)	55 (16%)	**< 0.001**	9 (6%)	30 (13%)	**0.02**
ICU time (hour)	49.2 ± 54.6	70.3 ± 129.1	**0.01**	50.5 ± 58.4	63.8 ± 122.1	0.21
Postoperative hospital stay (day)	14.7 ± 10.4	15.4 ± 8.4	**0.02**	15.2 ± 11.1	15.1 ± 7.4	0.17
Postoperative IABP	2 (1%)	5 (1%)	1.00	1 (1%)	3 (1%)	0.65
Postoperative mechanical support	1 (1%)	5 (1%)	0.43	1 (1%)	3 (1%)	0.65
30-days mortality	1 (1%)	15 (4%)	**0.01**	1 (1%)	6 (3%)	0.25

*CPB* cardiopulmonary bypass; *MI* myocardial infarction; *TIA* transient ischemic attack; Prolonged ventilation – artificial ventilation > 24 h; *ICU* intensive care unit; *IABP* intra-aortic balloon pump

Within each matched subgroup analysis of patients with mitral regurgitation, patients in the MINI group were more likely to have mitral valve repair than in the STERNOTOMY group (97% versus 77%; *p* <  0.001). Of note, in cases with leaflet prolapse, neochordae implantation technique was used more frequently in patients undergoing minimally invasive mitral valve repair (83% versus 53%, *p* <  0.001).

Two patients required conversion to median sternotomy (1.1%). In one patient because of severe aortic valve regurgitation due to injury of aortic valve. This was created by the stich for annuloplasty ring implantation. Another patient had severe pleural adhesions.

There were no differences between the two groups in terms of major complications, including myocardial infarction, cerebrovascular stroke, renal failure, atrial fibrillation, delirium, pneumonia, low cardiac output syndrome, and wound infections (Table 2). However, a minimally invasive approach was associated with a significantly lower rate of re-exploration for bleeding (*p* = 0.03) and lower postoperative blood loss (*p* = 0.02). Transfusion was less frequent after minimally invasive surgery than sternotomy (*p* = 0.001). Time to extubation was similar in both groups (*p* = 0.21); however, prolonged artificial ventilation (> 24 h) was less frequent in the MINI group (6% versus 13% in the STENOTOMY group; *p* = 0.02).

### Mid-term outcomes

**Fig. 1:**
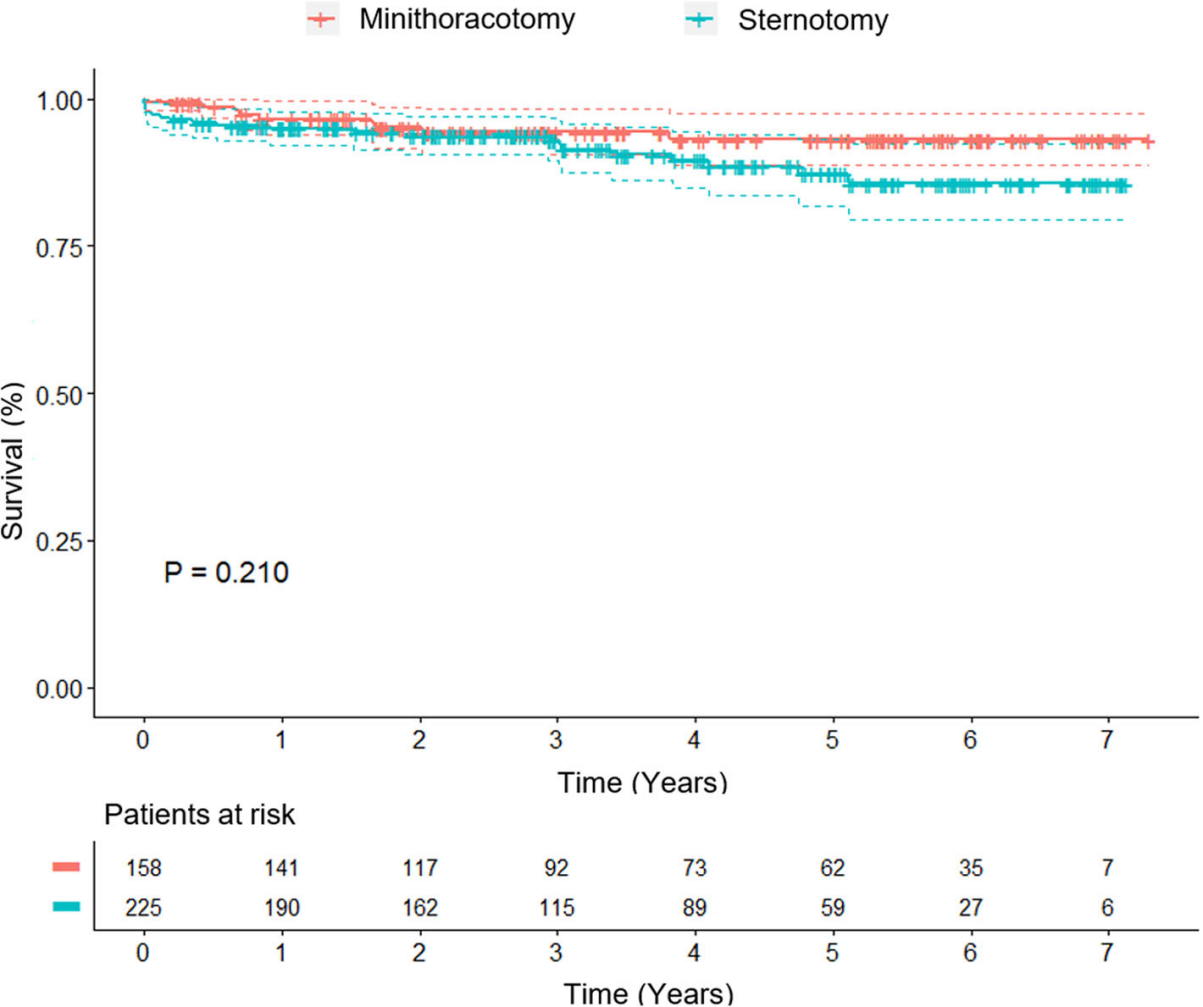
Kaplan-Meier estimate of survival of patients after propensity score matching. (95% confidence interval)

Survival data are illustrated in Fig. 1. The average follow-up was 1401.5 ± 768.9 days in the MINI group and 1232.1 ± 751.0 days in the STERNOTOMY group.

Kaplan–Meier estimates of mid-term survival in the matched patients, including operative deaths, for the MINI and STERNOTOMY groups were 96% versus 95% at 1 year and 93% versus 86% at 5 years, respectively, which showed no statistically significant difference (*p* = 0.21).

During the follow-up period, 9 patients (5.7%) died in the matched MINI group. The cause of death was assessed in all patients. Three of these patients died of cardiac-related causes, another 3 patients of stroke, 2 patients of malignant disease and 1 of other causes. In the matched STERNOTOMY group 19 patients (8.4%) died during the follow-up period. Eleven patients died from cardiac-related causes, 4 patients from malignant disease and 2 patients from stroke.

**Fig. 2:**
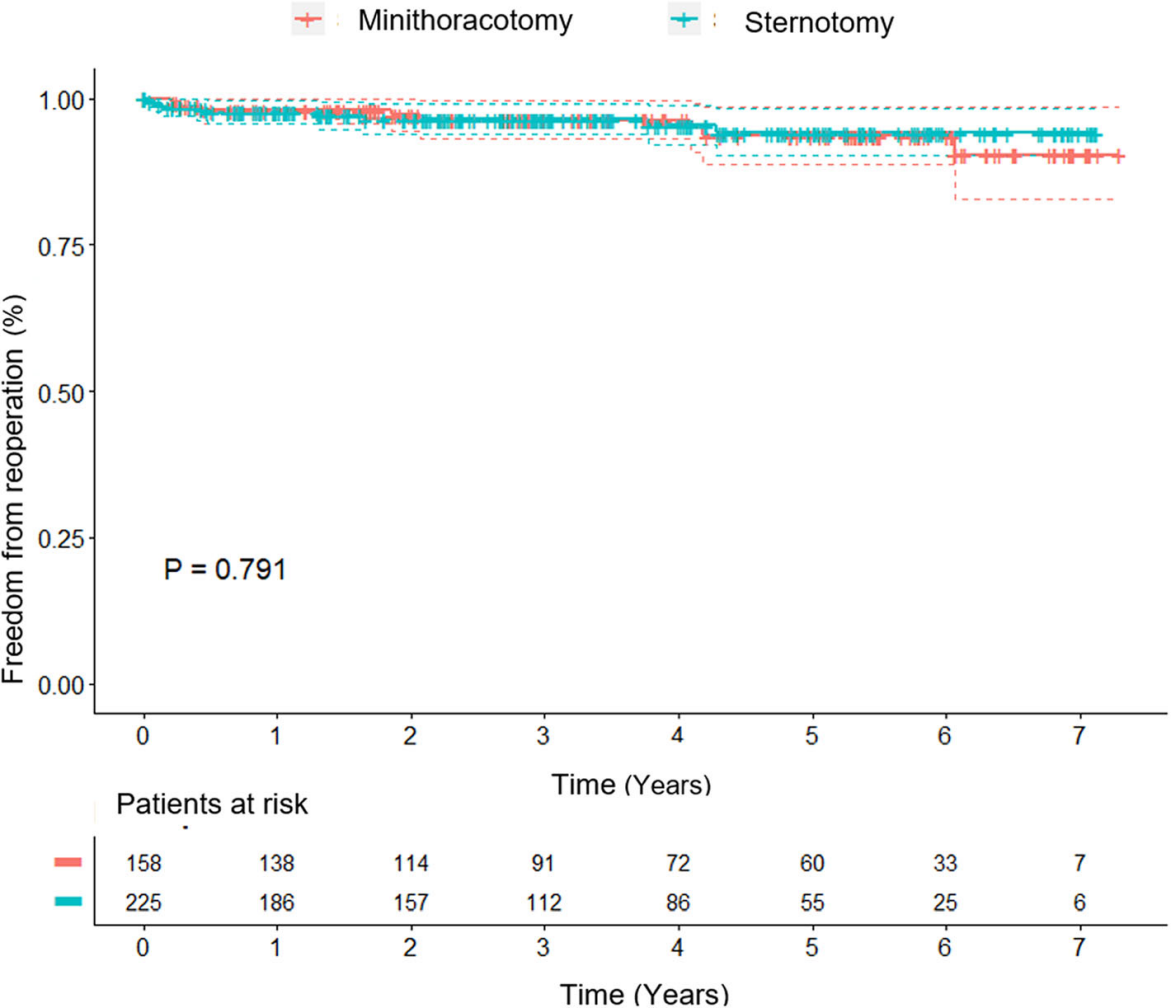
Kaplan-Meier estimate of freedom from mitral reoperation of patients after propensity score matching. (95% confidence interval)

The Kaplan–Meier plot for mitral valve reintervention in the matched groups is shown in Fig. 2. One- and five-year freedom from mitral reoperation were 97 and 95%, respectively, after minimally invasive surgery, and 97 and 94%, respectively, after median sternotomy, which showed no statistically significant differences (*p* = 0.79). In the matched MINI group 6 patients (3.8%) required reoperation. The indications for reoperation were failure of mitral valve repair in 4 patients (repeat reconstruction in 2 patients, mitral valve replacement in 2 patients) and infectious endocarditis in 2 patients. In the matched STERNOTOMY cohort, 9 patients (4%) required reoperation. Failure of mitral valve repair was observed in 7 patients (replacement in all patients) and infectious endocarditis in 2 patients.

### Hospital costs

Analysis of total hospital cost demonstrated equivalent values between the MINI and STERNOTOMY cohorts (*p* = 0.48).

There was a higher operative cost associated with minimally invasive approach than with full median sternotomy (*p* <  0.001). However, miniinvasive approach was associated with significantly lower blood product costs (p <  0.001). Higher operative cost of miniinvasive approach was offset by significantly lower postoperative costs for the minimally invasive cohort (*p* = 0.004). The distribution of costs is shown in Table 3.

**Table 3:** Matched hospital costs (EUR)

	Matched Patients		
	MINI	STERNOTOMY	*p*-value
Variable	n = 158	n = 225	
Total hospital costs	11,828 ± 6907	12,732 ± 99,936	0.48
Operative costs	5364 ± 1566	4778 ± 1920	**< 0.001**
Blood products costs	210 (0–393)	316 (109–545)	**< 0.001**
Postoperative costs	5054 (3993–6532)	5905 (4611–8304)	**0.004**

## Discussion

Minimally invasive approaches have been used for heart valve surgery with increasing frequency in recent decades. This has been driven primarily by technological advances and improvements in perfusion strategy. Patients are also increasingly requesting less-invasive mitral valve procedures, and referring physicians also recommend patients for minimally invasive procedures. However, surgeons have been slow to adopt MIMVS, and concerns regarding safety, an increased risk of stroke, and increased costs remain an issue in cardiac surgery centers. An analysis of the Society of Thoracic Surgeons data from 2011 to 2016 by Gammie et al. reported the frequency of all types of less-invasive mitral valve procedures to be approximately 23% [4]. According to the Annual Registry of German Society for Thoracic and Cardiovascular Surgery, the percentage of patients undergoing MIMVS increased from 13% in 2004 to 55% in 2018 [5, 6]. In Japan between 2008 and 2012, the frequency of MIMVS procedures increased from 6 to 16% [7].

In our propensity-matched study we were able to demonstrate that a minimally invasive approach is as safe as standard median sternotomy. Overall 30 day mortality in our matched cohorts did not differ between the two approaches (1% versus 3% (STERNOTOMY); *p* = 0.25). This principal finding of comparable mortality is supported by an excellent low mortality rate in the minimally invasive group, which was lower than the 2.6% ± 2.3% mortality predicted by EuroScore II. Moreover, despite longer operative and cardiopulmonary times, a minimally invasive approach was associated with a similar, or even lower, risk of adverse outcomes. Furthermore, the requirement for transfusions and postoperative blood loss was significantly better in the minimally invasive group. Therefore, the current patient cohort has demonstrated that MIMVS is a safe procedure, associated with a low incidence of intraoperative complications and excellent postoperative outcomes, which is in line with previous studies [8–11].

The quality of mitral valve repair is often an area of concern regarding MIMVS. However, previous studies have shown that MIMVS was associated with a mitral valve repair rate as high as 97% versus 77% through the conventional approach. Comparable results for the repair of complex mitral valve lesions has also been reported by Nasso et al. [12]. Even in the highly risky repair of Barlow valves, the success rate was comparable regardless of the approach used to repair the valve and was accompanied by a high degree of success. The durability of the repair was also seen to be equivalent.

The current study demonstrated comparable mid-term results with respect to postoperative survival and freedom from mitral valve reoperation between MIMVS and full sternotomy. These data suggest that clinical outcomes and the durability of mitral valve repair are not compromised by the use of a minimally invasive approach, which is also consistent with previous reports [8–10, 13–15].

Some reports have raised concerns about the potential for an increased risk of stroke associated with MIMVS. In 2010, the results of a meta-analysis conducted by the International Society of Minimally Invasive Cardiothoracic Surgery (ISMICS) showed comparable 30 day perioperative mortality, but a higher incidence of stroke was noted in the minimally invasive group (2.1% versus 1.2% in sternotomy group, *p* <  0.0001) [16]. However, it should be noted that this meta-analysis was primarily based on retrospective studies, and no propensity analysis was conducted. In addition, different aortic occlusion techniques and perfusion strategies were used. In a subgroup analysis of transthoracic clamping, the risk of stroke in patients undergoing the minimally invasive approach was similar to those undergoing median sternotomy. A higher incidence of stroke is usually explained by the difficulty of dearing the heart chambers by retrograde blood flow in the descending aorta, or by a longer duration of cardiopulmonary bypass. However, in propensity-matched comparisons published by Mkalaluh et al., Grant et al., and recently Paparella et al., no differences in the incidence of thromboembolic events were seen [9–11]. The incidence of stroke and transient neurological deficit in the matched groups of the current study showed no statistically significant difference, and this finding is consistent with the incidence of permanent neurologic deficit cited in previous publications. These promising results are due to a strict adherence to the exclusion criteria for MIMVS, which include atherosclerosis of the ascending aorta or severe atherosclerotic involvement of the pelvic and femoral arteries, which compromise the safety of the procedure. For these reasons, we recommend performing computed tomography angiography of the aorta and femoral arteries in all patients. Where atherosclerosis is evident, some authors advocate the use an alternative approach for cannulation, i.e., central cannulation of the ascending aorta or axillary artery.

A number of previous studies demonstrated significant clinical benefits associated with minimally invasive approaches in specific subgroups of patients that could benefit from alternative access. Santana et al. conducted a retrospective study of minimally invasive surgery in patients with chronic obstructive pulmonary disease [17]. Patients treated with a minimally invasive approach had a lower rate of hospital-related mortality than patients undergoing sternotomy (1% versus 5%, respectively) and a significantly lower incidence of all postoperative complications (30% versus 54%, respectively; *p* = 0.002). Finally, Holzhey et al. conducted a propensity-matched comparison to analyze the results of a less-invasive approach in elderly patients > 70 years of age [18]. No differences were seen in 30 day mortality (7.7% versus 6.3% in the sternotomy group, *p* = 0.82) or combined cardiac and cerebrovascular complications (11.2% versus 12.6%, respectively; *p* = 0.86).

In general, minimally invasive approaches are considered to be more expensive than traditional approaches. Nevertheless, as we have demonstrated in the current study, MIMVS can be performed with equivalent total hospital cost and outcomes to those seen in patients who underwent the same procedure through a full median sternotomy. The current study also showed that a minimally invasive approach was associated with fewer transfusions, a lower rate of re-exploration for bleeding, less frequent prolonged artificial ventilation, and a trend toward shorter ventilation time, which translated into lower blood product and ancillary costs. These cost savings were offset by higher surgical costs. The use of minimally invasive soft tissue retractors and perfusion cannulas also contributes to the higher surgical costs. Our findings corroborate the results of previous studies that evaluated the economic impact of minimally invasive approaches in mitral valve repair. Atluri et al. evaluated resource utilization, including the cost of minimally invasive approaches, in mitral valve repair surgery. Although they used more expensive endoaortic cross-clamps, total hospital costs were seen to be similar to conventional approaches [19]. Overall, these reports, in addition to the findings of the current study, demonstrate equivalent cost between minimally invasive and conventional approaches [19, 20].

This study has several limitations, including the retrospective study design with inherent bias in data collection, in addition to the fact that it was conducted in a single center. As with any retrospective analysis, there is inherent selection bias in the surgeon’s decision to perform a given surgical approach. The results of studies using propensity score matching are influenced by the number and selection of variables included in the propensity score model. The patients in the study may still be unbalanced with respect to unmeasured variables. Finally, a longer period of follow-up would be required to report long-term survival and treatment success.

## Conclusion

A minimally invasive approach is a feasible, safe, and reproducible technique with excellent short-term outcomes, and comparable mid-term outcomes and efficacy to conventional sternotomy for patients undergoing mitral valve surgery. In addition to improved cosmetic results, a minimally invasive approach provides equally durable results to standard sternotomy. Finally, the total hospital costs associated with MIMVS are equivalent to conventional median sternotomy. Based on these findings, a minimally invasive approach should be considered for all patients who require mitral valve surgery.

## Data Availability

The dataset used and/or analysed during the current study are available from corresponding author on reasonable request.

## Abbreviations

MIMVS: Minimally invasive mitral valve surgery
ISMICS: International society of minimally invasive cardiothoracic surgery
